# Oxy-Phosphate

**Published:** 1885-07

**Authors:** E. G. Betty

**Affiliations:** Cincinnati


### THE DENTAL REGISTER.

Vol. XXXIX.] JULY, 1885. [No. 7.

### COMMUNICATONS.

### Oxy-phosphate.

BY E. G. BETTY, D.D.S., CINCINNATI.

Read before the Mad River Valley Dental Society, Dayton, May 19, 1885.

Under the head of "Different Materials for and Methods of Filling Teeth," it may not be amiss to say a few words about our very good servant, oxy-phosphate of zinc.

This substance, as all are aware, is the result of a chemical union between a base and an acid; but with that part of it we will have nothing to do to-day. I am daily becoming more convinced that this material is of more service than we imagine in this that, it enables us to ultimately save more "aching" teeth than we could were we still dependent upon the oxy-chlorides. It is my custom to fill *all* teeth with it in which the decay has been so extensive as to nearly expose the pulp, those in which it *is* exposed, and dead teeth that have undergone conservative treatment. In case the decay has not exposed the pulp, the debris is thoroughly removed and the cavity filled with a bolus of the phosphate mixed as dry as possible; the surplus is then removed and the filling covered with wax or parafine, or it may be given a smooth surface by polishing with heated talc. Should the pulp be exposed, remove the decay, cover the pulp with some kind of varnish—comp. tinct. benzoin is most excellent, flow over it a little of the phosphate prepared very thin, and when hardened, trim and fill as in the first case. Dead teeth are also to be filled with a stiff bolus. The main point in the use of the phosphate, if you wish to secure full service from it, is to allow the filling to remain as long as it will last, which, in very many cases, is about two years. Should it not remain so long, refill. The object of this is to give the tooth a *long rest*, the longer the better; at the end of a year and a half or two years, large operations with cohesive foil will be borne by the tooth so treated with but a slight chance of producing either death of the pulp in the one case, or recurrence of periosteal inflammation in the other.

A writer, in the *Independent Practitioner* for May, recommends that a small quantity of iodoform be mixed with the thin oxyphosphate to be flowed on exposed pulps, or immediately over the thin dentine covering one almost exposed. Though I have not tried this, yet I am inclined to think the principle is good.

